# A Genetic Polymorphism (rs17251221) in the Calcium-Sensing Receptor Gene (*CASR*) Is Associated with Stone Multiplicity in Calcium Nephrolithiasis

**DOI:** 10.1371/journal.pone.0025227

**Published:** 2011-09-22

**Authors:** Yii-Her Chou, Peng Yeong Woon, Wei-Chiao Chen, Yu-Wen Hsu, Jer-Ming Chang, Daw-Yang Hwang, Yi-Ching Chiu, Ho-Chang Kuo, Wei-Pin Chang, Ming-Feng Hou, Mu-En Liu, Jan-Gowth Chang, Wei-Chiao Chang

**Affiliations:** 1 Department of Urology, College of Medicine, Kaohsiung Medical University, Kaohsiung, Taiwan; 2 Department of Urology, Kaohsiung Medical University Hospital, Kaohsiung, Taiwan; 3 Department of Medical Genetics, College of Medicine, Kaohsiung Medical University, Kaohsiung, Taiwan; 4 Cancer Center, Kaohsiung Medical University Hospital, Kaohsiung, Taiwan; 5 Department of Healthcare Management, Yuanpei University, HsinChu, Taiwan; 6 Department of Medical Research, Kaohsiung Medical University Hospital, Kaohsiung, Taiwan; 7 Department of Molecular Biology and Human Genetics, Tzu Chi University, Hualien, Taiwan; 8 Division of Nephrology, Department of Medicine, Kaohsiung Medical University Hospital, Kaohsiung, Taiwan; 9 Division of Allergy, Immunology and Rheumatology, Chang Gung Memorial Hospital-Kaohsiung Medical Center, Kaohsiung, Taiwan; 10 Division of Cardiology, Department of Pediatrics, Chang Gung Memorial Hospital-Kaohsiung Medical Center, Kaohsiung, Taiwan; 11 Department of Surgery, College of Medicine, Kaohsiung Medical University, Kaohsiung, Taiwan; 12 Center for Resources, Research and Development, Kaohsiung Medical University, Kaohsiung, Taiwan; Tor Vergata University of Rome, Italy

## Abstract

Calcium nephrolithiasis is one of the most common causes of renal stones. While the prevalence of this disease has increased steadily over the last 3 decades, its pathogenesis is still unclear. Previous studies have indicated that a genetic polymorphism (rs17251221) in the calcium-sensing receptor gene (*CASR*) is associated with the total serum calcium levels. In this study, we collected DNA samples from 480 Taiwanese subjects (189 calcium nephrolithiasis patients and 291 controls) for genotyping the *CASR* gene. Our results indicated no significant association between the *CASR* polymorphism (rs17251221) and the susceptibility of calcium nephrolithiasis. However, we found a significant association between rs17251221 and stone multiplicity. The risk of stone multiplicity was higher in patients with the GG+GA genotype than in those with the AA genotype (chi-square test:*P* = 0.008;odds ratio  =  4.79;95% confidence interval, 1.44–15.92;Yates' correction for chi-square test:*P* = 0.013). In conclusion, our results provide evidence supporting the genetic effects of *CASR* on the pathogenesis of calcium nephrolithiasis.

## Introduction

Nephrolithiasis is a worldwide health problem that affects almost all populations. In Taiwan, its prevalence is approximately 9.6% (14.5% in males and 4.3% in females) [1]. The cause of nephrolithiasis is still unclear. Environmental factors such as lifestyle, obesity, and dietary habits seem to be involved in its development [2], but its pathogenesis may also be influenced by hormonal, genetic, or anatomical factors [3]. Previous studies have indicated that genetic polymorphisms of the calcium-sensing receptor (*CASR*), vitamin D receptor, and osteopontin genes are highly associated with stone formation [4]. Therefore, genetic polymorphisms are important for susceptibility to this disease [4].

Hypercalciuria is one of the main risk factors for nephrolithiasis. Most patients with nephrolithiasis have calcium-containing stones [5]. Therefore, the physiological processes that influence calcium delivery to the kidney may influence stone formation. Perturbations in calcium homeostasis are considered the most important risk factors for calcium nephrolithiasis [6]. The CASR located in the plasma membrane of renal tubule cells is a vital regulator of cellular calcium homeostasis [7]. A previous study has shown that an inactivating mutation of *CASR* causes familial hypocalciuric hypercalcemia (FHH), which leads to life-long hypercalcemia [8]. In addition, a *CASR* genetic polymorphism (rs17251221) is strongly associated with serum calcium concentration in individuals of European and Indian-Asian descent [9]. However, the genetic effect of *CASR* (rs17251221) on susceptibility to nephrolithiasis is still unclear.

The aim of the present study was to investigate whether the *CASR* polymorphism (rs17251221) is associated with the development of calcium nephrolithiasis. The number of renal stones is related to the disease activity of nephrolithiasis; therefore, we investigated the association between the *CASR* genetic polymorphism and the number of renal stones in patients with nephrolithiasis.

## Materials and Methods

### Subject recruitment

We conducted a case–control study from February 2009 to December 2010 in Kaohsiung Medical University Hospital, Kaohsiung, Taiwan. In total, 189 patients with calcium nephrolithiasis and 291 controls were included in the study. Calcium nephrolithiasis was diagnosed on the basis of ultrasonographic and radiographic findings. A 20-mL whole blood sample and a one-spot midstream urine sample from the first voided urine were collected from all subjects after they had fasted overnight. Blood and urine samples were used to measure total calcium, phosphate, uric acid, and creatinine levels. All urinary parameters were corrected by using urinary creatinine levels. If stone specimens were removed by surgery or obtained after medical treatment or shock-wave lithotripsy, infrared spectroscopy (Spectrum RX I Fourier Transform-Infrared System; Perkin Elmer, Shelton, CT, USA) was used to confirm the composition of calcium-containing stones [5]. Patients were excluded if they had a history of chronic urinary tract infection, renal failure, chronic diarrhea, gout, renal tubular acidosis, primary and secondary hyperparathyroidism, or cancer. We also excluded anyone who had regularly taken diuretics, vitamin D, or calcium supplements more than or equal to 1 time per week within the 6 months leading up to the diagnosis of calcium nephrolithiasis or the interview, because these substances may have confounded our results. Further, in the stone number analysis, patients with a single stone were grouped, whereas patients with stone numbers over than one were grouped. The 291 controls had normal urinalysis results, no history of familial urinary stone disease, and no history of renal calcification at health screening. All study subjects were residents of southern Taiwan and provided the required written consent forms. The study protocol conformed to the Declaration of Helsinki, and the study was approved by the Institute Review Board of Kaohsiung Medical University Hospital.

### DNA extraction

Genomic DNA was extracted from whole blood samples by using a standard protocol. Whole blood samples from patients and controls were centrifuged at 3000 rpm for 10 min at 4°C. Buffy coat was isolated from the blood samples. After lysing the red blood cells in a lysis buffer, the samples were mixed with a cell lysis buffer for several days. Protein precipitation solution was added to precipitate the proteins. Finally, total genomic DNA was isolated by precipitation with 95% isopropanol and 80% alcohol.

### Genotyping CASR polymorphisms

A single nucleotide polymorphism (SNP) in *CASR* (rs17251221) was selected on the basis of research findings reported by O'Seaghdha *et al* [10]. The TaqMan Allelic Discrimination Assay (Applied Biosystems, Foster City, CA, USA) was used for genotyping. Briefly, the polymerase chain reaction (PCR) was performed using the ABI7900 Thermal Cycler. The thermal cycle-sequencing conditions were as follows: initial denaturation at 95°C for 10 min, followed by 45 cycles of denaturing at 95°C for 15 s and annealing and extension at 60°C for 1 min. Data was measured and analyzed using the System SDS software version 1.2.3.

### Statistical analysis

The distribution of genotypes was in Hardy–Weinberg equilibrium (*P*>0.05) for both patients and controls. The statistical differences between the patient and control groups or between the multiple and single stone-episode groups in genotype and allele frequency were analyzed by the chi-square test. Under certain circumstances, Yates' correction for the chi-square test was also applied for a more stringent analysis. The relationship between kidney stone diseases and multiplicity and *CASR* polymorphisms was assessed by the odds ratio (OR). Quantitative variables were expressed as the mean ± standard deviation. The *t*-test was used to compare the differences between the means of continuous variables. A *P* value < 0.05 was considered statistically significant. Statistical analyses were performed using SPSS, version 14.0 (SPSS Int., Chicago, IL, USA).

## Results

### Lack of association between the CASR rs17251221 polymorphism and the susceptibility to calcium nephrolithiasis

In total, 480 subjects were enrolled in this study, including 189 patients with calcium nephrolithiasis and 291 controls. Men accounted for 66.1% of the patients and 47.1% of the controls. The age of the subjects ranged from 19 to 88 years. The mean age of the patients was 52.6±12.6 years; the controls had a similar age distribution.

The genotype frequencies of the cases and controls were in accordance with the Hardy–Weinberg equilibrium. Table 1 shows the genotype frequencies of *CASR* polymorphism among subjects. Since there was only 1 patient with the GG genotype, we classified patients into 2 groups based on their genotypes, namely, the GG+GA and AA groups. There were no significant differences in the genotypes or allele frequencies of the SNP between the controls and patients with calcium nephrolithiasis.

**Table 1:** frequencies for *CASR* rs17251221 and nephrolithiasis susceptibility.

	Genotype	Case (%)(n = 189)	Control (%)(n = 291)	Allele	Case (%)(n = 189)	Control (%)(n = 291)	Genotype*P* value	Dominant*P* value	Recessive*P* value	Allelic*P* value
rs17251221	GG	1 (0.5%)	0 (0.0%)	G	15 (4.0%)	18 (3.1%)	0.440	0.600	0.214	0.467
	AG	13 (6.9%)	18 (6.2%)	A	363 (96.0%)	564 (96.9%)				
	AA	175 (92.6%)	273 (93.8%)							

### The genetic polymorphism (rs17251221) in CASR is significantly associated with stone multiplicity in nephrolithiasis patients

We further evaluated whether an association existed between *CASR* and the characteristics of kidney stone patients, including age, gender, number of stones, and frequency and family history of stones. As shown in Table 2, the G allele of the polymorphism rs17251221 is a risk allele that seems to have a dominant genetic effect over the A allele, because the risk for stone multiplicity was higher in patients in the GG+GA genotypic group than in the AA genotypic group (*P* = 0.008; OR = 4.79; 95% confidence interval, 1.44–15.92; Table 2). Similarly, a significant association between rs17251221 and stone multiplicity was also observed when we performed the more stringent Yates' chi-square test (*P* = 0.013; Table 2).

**Table 2:** Association analysis between *CASR* rs17251221 and subgroups of 189 patients with kidney stone.

	*CASR* genotype		*P* value	OR(95% CI)	*P* value^a^
	GG+AG(n = 14)	AA(n = 175)			
Age, Mean ± SD	51.7±10.0	52.6±12.8	0.798		
Gender, Male (%)	8 (57.1%)	117 (66.9%)	0.559		
Stone numbers (%)					
Multiple	10 (71.4%)	60 (34.3%)	**0.008** *	4.79(1.44–15.92)	**0.013** *
Single	4 (28.6%)	115 (65.7%)			
Stone frequency (%)					
Recurrence	7 (50.0%)	62 (35.4%)	0.387		
Non- recurrence	7 (50.0%)	113 (64.6%)			
Family history (%)					
Positive	3 (21.4%)	45 (25.7%)	1.000		
Negative	11 (78.6%)	130 (74.3%)			

*Significant (*P*<0.05) values are in bold.

**^a^Yates' correction for chi-square test.**

### Lack of association between rs17251221 polymorphism of CASR and biochemical parameters in nephrolithiasis patients

To understand the relationship between the rs17251221 polymorphism of *CASR* and clinical risk factors, we analyzed the biochemical data, including the levels of urinary and serum creatinine, uric acid, and calcium. None of the clinical parameters reached a nominal significance level of 0.05 (Table 3), and subset analysis on the cases did not yield any significant results (Table 4).

**Table 3:** Association analysis between *CASR* rs17251221 and clinical biochemical data in patients with calcium kidney stone.

SNP	Genotype	UrineCreatinine(mg/dl)	UrinepH	UrineCalcium(mg/dl)	UCa/UCr^a^	UrineUric Acid (mg/dl)	UUA/UCr^b^	SerumCreatinine(mg/dl)	SerumCalcium(mg/dl)	SerumUric Acid(mg/dl)
rs17251221	GG+AG	65.6±50.0	5.9±0.6	9.0±3.4	0.2±0.1	29.2±19.7	0.9±1.5	1.0±0.3	8.9±0.5	6.0±1.8
	AA	85.2±59.6	6.1±0.6	11.4±7.5	0.2±0.1	39.0±25.1	0.5±0.2	1.1±0.8	8.7±0.7	6.3±1.6
	*P* value	0.2505	0.2481	0.0713	0.6259	0.1706	0.3686	0.2784	0.5281	0.6277

a Urinary calcium-Creatinine ratio (UCa/UCr).

b Urinary uric acid-Creatinine ratio (UUA/UCr).

**Table 4:** Association analysis between *CASR* rs17251221 and subgroups of biochemical data in patients with calcium kidney stone.

	*CASR* genotype		*P* value	OR(95% CI)
	GG+AG	AA		
Serum Uric Acid (%)	N = 13	N = 156		
>7.2 (mg/dl)	3 (23.1%)	42 (26.9%)	0.763	0.81(0.21–3.10)
≤7.2 (mg/dl)	10 (76.9%)	114 (73.1%)		
Serum Calcium (%)	N = 13	N = 156		
>10.2 (mg/dl)	0 (0.0%)	2 (1.3%)	0.681	
≤10.2 (mg/dl)	13 (100.0%)	154 (98.7%)		
Serum Creatinine (%)	N = 13	N = 156		
>1.3 (mg/dl)	3 (23.1%)	28 (18.0%)	0.646	1.37(0.35-5.31)
≤1.3 (mg/dl)	10 (76.9%)	128 (82.0%)		
UCa/UCr^a^ (%)	N = 10	N = 133		
≥0.2	3 (30.0%)	40 (30.1%)	0.996	1.00(0.25–4.05)
<0.2	7 (70.0%)	133 (69.9%)		

a Urinary calcium-Creatinine ratio (UCa/UCr).

## Discussion

Nephrolithiasis is one of the most common urologic diseases in the developed countries. Chemical composition analysis of urinary stones has indicated that calcium oxalate stones are the most common and occur in approximately 75% to 80% of cases [11]. Consequently, factors that affect the serum levels of calcium ion are important risk factors for calcium nephrolithiasis [12]. CASR is a G-protein coupled receptor, which senses and sets the extracellular calcium ion levels by regulating renal calcium excretion and controlling parathyroid hormone secretion from the parathyroid glands [13]. Evidence from gain- or loss-of-function mutations in the *CASR* gene indicate that this gene plays a critical role in calcium ion homeostasis [13]. In the kidney, CASR prevents the reabsorption of divalent cations in the cortical thick ascending limb and triggers the inhibitory actions of hypercalcemia on the urinary-concentrating mechanism, subsequently leading to the prevention of kidney stone formation [14], [15]. To our knowledge, this is the first study to report a *CASR* polymorphism (rs17251221) associated with the risk of stone multiplicity in calcium nephrolithiasis.

Nephrolithiasis research has focused on biochemical or metabolic alterations in local urinary constituents leading to stone formation. However, abnormalities in the chemical composition of urine alone do not explain many aspects of nephrolithiasis urinary stone disease [11]. Recent evidence from genome-wide association studies suggests that the rs17251221 polymorphism in *CASR* is strongly associated with total serum calcium levels [9], [10]. Indeed, a genome-wide meta-analysis showed that common *CASR* variants modulate serum calcium levels in the adult general population [9], [10]. In agreement with the findings of the above-mentioned study and those of other studies [4], [10], [16], [17], [18], we have observed an association between the rs17251221 minor G allele and the disease activity of calcium nephrolithiasis. However, we did not observe a significant association between higher levels of serum or urine calcium and this SNP in a Taiwanese population. We attribute this finding to the low statistical power of using a limited number of subjects (only 169 cases with clinical biochemical data). Additionally, when testing for the effects of the *CASR* genotype on clinical biochemical parameters we did not use a control group. The availability and use of data from the controls would have strengthened our observation. Another possible explanation for our results is the different genetic backgrounds of the populations and the phenotypic heterogeneity of nephrolithiasis. Our findings still need to be replicated in a larger sample size.

*CASR* gene mutations have been implicated in several rare familial genetic disorders. For instance, the gain-of-function mutations E128A (Glu128Ala; rs121909260; MIM #601199.0004) and T151M (Thr151Met; rs104893694; MIM #601199.0012) cause an autosomal dominant form of hypocalcemia (MIM #146200), whereas R990G (Arg990Gly; rs1042636) is a gain-of-function mutation that was reported to predispose patients to primary hypercalciuria and kidney stone disease [10], [16]. Interestingly, the R990G variant increases patient susceptibility to primary hypercalciuria in patients with kidney stones [16]. In contrast, loss-of-function mutations in FHH result in the inactivation of *CASR* and subsequent hypercalcemia and hypermagnesemia [19]. O'Seaghdha *et al* have indicated that the minor G allele of rs17251221 was associated with higher serum calcium and magnesium levels and suggested that the causal G variant may underlie a reduction in CASR activity [10]; this finding also suggested a role of CASR in regulating extracellular calcium concentrations. However, a search of the Ensembl (University of California, Santa Cruz) genome browser and ConSite databases for regulatory elements around the genomic region corresponding to rs17251221 did not reveal any functional regulatory elements. O'Seaghdha *et al* pointed out that there was another coding SNP in this gene, rs1801725 (A986S), which has been detected among families with FHH and in the general population [10]. Thus, further studies to test the association between rs1801725 and nephrolithiasis may provide a better understanding of this disease.

In conclusion, our data showed a significant association between rs17251221 and stone multiplicity in nephrolithiasis patients, suggesting that rs17251221 is as a susceptibility marker for stone multiplicity in nephrolithiasis. Moreover, this is the first evidence that the risk of developing renal stones was greater in nephrolithiasis patients having the G allele of rs17251221 than those having the A allele.
